# Oral health knowledge, attitudes, and practices of paediatric nurses caring for hospitalized children

**DOI:** 10.3389/fdmed.2024.1426697

**Published:** 2024-06-19

**Authors:** Ashley Fletcher, Shauna Hachey, Tracy Doyle

**Affiliations:** ^1^ Department for Dalhousie University Faculty of Dentistry, Halifax, NS, Canada; ^2^ School of Dental Hygiene, Dalhousie University Faculty of Dentistry, Halifax, NS, Canada; ^3^ Healthy Populations Institute, Halifax, NS, Canada; ^4^ Division of Pediatric Dentistry, Dalhousie University Faculty of Dentistry, Halifax, NS, Canada; ^5^ Paediatric Dentistry, IWK Health, Halifax, NS, Canada

**Keywords:** integrated oral care, pediatrics, nursing, interprofessional collaboration, hospital, inpatient care

## Abstract

**Objectives:**

Nurses are well positioned to provide oral care to hospitalized children. This study explores pediatric hospital nurses’ knowledge, attitudes, practices and perceived barriers to providing oral care.

**Methods:**

Using a descriptive cross-sectional design, previously validated surveys were adapted based on input from key stakeholders and administered to all nurses and staff providing patient care on inpatient units (*N* = 239) of a pediatric hospital.

**Results:**

The survey response rate was 40% (*N* = 96), providing a margin of error of 7.59% (95% C.I.). Most participants were unaware that caries is infectious (51%, *n* = 49) and caries-producing bacteria is transmissible (35%, *n* = 34). The majority (57%, *n* = 52) of participants did not recall oral care content within their formal education or oral care continuing education (88%, *n* = 81), despite high interest (87%, *n* = 80). Oral care was rated by most as a priority (85%, *n* = 81), yet the majority (74%, *n* = 69) believed it is under performed. More nurses with 6 or more years of experience placed a high or very high value on prioritizing oral health (p = 0.005). Furthermore, most nurses do not assess oral health on admission (63%, *n* = 60), routinely incorporate oral health into the care plan (45%, *n* = 43), or document oral care (60%, *n* = 56). Commonly reported barriers include lack of patient cooperation, medical status, and competing needs.

**Conclusions and outcome:**

Despite nurses valuing the importance of oral care and their willingness to learn, oral care practices are lacking, and barriers exist. Future investigation is required to further explore the findings related to barriers to care and lack of practice. These results and future findings will be used to guide institutional oral care policy and education.

## 1 Introduction

The oral cavity is the entrance point for external pathogens to enter the human body and by nature, the oral cavity is equipped to protect the body from these pathogens when healthy. A healthy oral microbiome provides resistance to pathogenic invaders, and when there is a disruption to the composition, activity, and function of the microbiome, oral disease occurs. Common oral diseases include dental caries and gingival diseases (1). Factors influencing the microbiome include, but are not limited to, biofilm maturation, host environment, oral hygiene habits, medications, genetics etc.

Dental caries is an infectious, multifactorial disease influenced by the social determinants of health and barriers to accessing oral health care (2). Severe dental caries can have negative systemic and psychological implications, and impair children's growth and development (3, 4). In Canada, it is estimated that 2.26 million school days are lost each year due to oral disease (5–7) and oral rehabilitation accounts for one third of all day surgeries performed on children between ages one to five (8). Similar statistics are found in the United States, where children with poor oral health were three times more likely to miss school as a result of dental pain, resulting in a poorer school performance compared to their counterparts (9).

Oral health is integral to systemic health and inverse relationships exist (10). Medically compromised children with significant co-morbidities and special health care needs are particularly at risk for poor oral health (11–14). Due to the numerous competing medical needs among this population, oral health is typically less prioritized, leading to missed dental appointments or neglect (11, 15). Furthermore, many of these children are burdened with social determinants of health which result in compromised oral and general health outcomes. Medically compromised children often spend time as hospital inpatients and are likely to present with oral disease (4, 6, 11). To further increase the risk of oral disease among children who are hospitalized, normal practices and routines are often disrupted due to immediate and urgent medical concerns. Often, oral hygiene is overlooked due to prioritizing immediate medical needs. As a result, oral health care needs for many children with special health care needs remain unmet (11, 16, 17).

Nurses and care team assistants (nursing staff) are at the forefront of delivering daily patient care in hospitals, and as such, are well positioned to contribute to the oral health, and in turn overall health of hospitalized children. For example, daily oral hygiene based on a validated oral health assessment and using a step-by-step daily care plan has shown to reduce ventilation-associated or hospital-acquired pneumonia in patients (14, 18). While nurses note the importance of oral health (19, 20), there is a need to improve nurses’ attitudes, knowledge, and practices regarding oral care (19–21). Martins et al. reported that 34.8% (*n* = 46) of children did not receive daily oral care during their hospital stay, and 97.8% (*n* = 46) of parents received no oral health information. Almost half of the children who participated in this study (47.8%, *n* = 46) experienced dental caries, and 58.7% (*n* = 46) were on a liquid medication containing sucrose, thus increasing their dental caries risk (17). Furthermore, there is limited evidence citing the impact of continuing education in healthcare institutions and the availability of guidelines for nursing staff with regards to oral care (21). There is also limited documentation of oral health care education in nursing curricula, however, support for the addition of didactic and clinical oral health care components exists (22, 23). Moreover, there is limited evidence pertaining to the North American context.

The purpose of the study is to investigate pediatric nursing staff's:

(1) Knowledge related to oral health
(2) Attitudes related to the provision of oral care for their patients
(3) Current oral health practices in daily patient care
(4) Perceived barriers to providing oral care.

## 2 Methods

This study was conducted in a tertiary care pediatric hospital in Canada. A cross-sectional descriptive study design was used to develop and administer surveys to frontline nurses regarding oral health attitudes, knowledge and practices. Survey questions were identified and adapted from previously validated surveys (19–21) and administered to nurses in various hospital settings. Survey questions were modified based on input from the nurse leaders of each inpatient unit in the tertiary care pediatric hospital. Discussions with the nurse leaders provided insight of current oral care practices, the availability of oral hygiene aides, orientation and continuing oral care education offered to new and existing staff, and perceived barriers to the provision of oral care. Text boxes to allow for open-ended responses were also provided.

Practicing registered nurses (RNs), licensed practical nurses (LPNs) and care team assistants (CTA's) who provide patient care within inpatient pediatric units were invited to participate. The recruitment goal was 148 participants, to provide a 5% margin of error (95% C.I.). Staff from the Intensive Care Unit; Medical Unit; Medical, Surgical and Neurosciences Unit; Nephrology, Hematology and Oncology Unit; and the Psychiatric Inpatient Unit were asked to participate. Excluded from the study were inpatient units for neonates and newborns, and all outpatient units (such as Ambulatory Clinics, Day Surgery and Emergency Departments). These units were excluded because of the unique oral care needs of newborns and outpatients, compared to inpatient oral care for dentate patients. The survey was initially piloted with one RN and one LPN to ensure the questions were appropriate and read as intended. Implied consent was provided by answering the survey questions, which was clearly outlined in the consent form. The study received institutional Research Ethics Board approval. (File no. 1026922). The institution does not currently have a universally adopted oral health guideline for hospitalized patients.

The survey was conducted online through Opinio^TM^ software. Participant responses remained anonymous, and participation was limited to one time. Between September 2021 and January 2022, participant recruitment was conducted through the institutional email listserv and posters in each of the included inpatient units. Separate from survey responses, participants were given the option to provide their contact information to be eligible to win one of two power toothbrushes, drawn at the halfway point and the end of data collection. The participant's contact information was not linked to their survey responses in any way.

The data were exported from Opinio^TM^ software™ to SPSS™ V29 statistical software for analysis. Descriptive statistics were calculated for each variable. A series of bivariate inferential comparisons were run using the chi-square test to examine differences between categorical variables. Missing data was excluded from this analysis. The Chi-square test was used to determine whether knowledge (using responses to the knowledge test questions), practices (i.e., oral assessment, advising on oral implications of medication, care planning and documentation) and prioritization of oral health varied by age, experience, exposure to formal oral health education or unit. Cramer's V (value) was used to determine the magnitude of the association between the categorical variables.

## 3 Results

### 3.1 Demographics

Among the 239 RN's, LPN's and CTA's, 96 participated in this study, resulting in a response rate of 40%. Participants included 91 RNs and 5 LPNs (*N* = 96); the calculated margin of error was 7.59 with 95% confidence interval. No CTA's participated in the survey; therefore, the results reflect the perspectives of the nursing staff. Each of the inpatient units were represented: Psychiatric Inpatient Unit (15%, *n* = 14), Intensive Care Unit (24%, *n* = 23), Medical Unit (28%, *n* = 27), Medical, Surgical and Neurosciences Unit (11%, *n* = 11), and Nephrology, Hematology and Oncology Unit (21%, *n* = 20). Most participants had less than 15 years of work experience: 0–5 years (43%, *n* = 41), 6–15 years (32%, *n* = 31), 16–30 years (19%, *n* = 18), and 30+ years (6%, *n* = 6). Lastly, most participants were 45 years of age or younger: 20–30 years (42%, *n* = 40), 31–45 years (41%, *n* = 39), 46–60 years (14%, *n* = 13), 60+ years (2%, *n* = 2) (Table 1).

**Table 1:** Demographic profile (*N* = 96).

	n	%
Age (years)		
0-30	39	40.63%
31-45	39	40.63%
46-60	15	15.63%
60+	2	2.08%
Prefered not to specify	1	1.03%
Profession		
Registered Nurse	91	94.79%
Licensed Practical Nurse	5	5.21%
Years of Experience		
0-5	41	42.71%
6-15	31	32.29%
16-30	18	18.75%
31+	6	6.25%
Unit		
Intensive Care Unit	23	23.96%
Medical Unit	27	28.13%
Medical, Surgical and Neurosciences Unit	11	11.46%
Nephrology, Hematology and Oncology Unit	20	20.83%
Psychiatric Inpatient Unit	14	14.58%
Prefered not to specify	1	1.04%

### 3.2 Knowledge

Only half of the participants (51%, *n* = 49) were aware that caries is an infectious disease, and the majority (65%, *n* = 62) did not know that caries-causing bacteria are transmissible. Just over half (52%, *n* = 50) of the participants were aware that a smear, or rice grain size of fluoridated toothpaste is an appropriate amount for a child under the age of 3 (24). A pea size amount of fluoridated toothpaste is appropriate for children over 3 years of age and able to spit (24). A larger percentage (76%, *n* = 73) of participants were aware of this guideline for older children (Table 2).

**Table 2:** Descriptive analysis of survey results.

	n	%
Education (Responded yes)		
Oral health content was included in formal nursing education	40	41.67%
The oral health content within formal nursing education was adequate	23	23.96%
Interested in obtaining education in oral health	80	83.33%
Knowledge (Chose the correct multiple choice answer)		
Cavities are an infectious disease	49	51.04%
Cariogenic bacteria is transmissible	34	35.42%
Children under 3 or unable to spit can use a smear (i.e., the size of a grain of rice) amount of fluoridated toothpaste	50	52.08%
Children over 3 and able to spit can use a pea sized amount of fluoridated toothpaste	73	76.04%
Attitudes/Beliefs (Agreed or strongly agreed)		
Oral health is a significant component of overall health	93	96.88%
Daily mouthcare is equally important for all patients	89	93.68%
A tool to assist in mouthcare planning would be helpful	36	83.72%
Nurses are responsible to ensure daily mouthcare is delivered to patients either by the patient/caregiver or themselves	76	81.72%
Patient/caregiver's are responsible to ensure daily mouthcare is delivered by either asking for assistance or completing it themselves	71	76.34%
Mouthcare is performed as often as neccessary	25	26.88%
Comfort (Agreed or strongly agreed)		
Comfortable identifying healthy and unhealthy findings in the mouth and bring concerns to the attention of the care team	71	74.74%
Comfortable performing daily mouthcare regardless of a patient's medical condition	74	78.72%
Practices (Responded yes)		
Patient's oral health status is routinely assessed on admission	26	27.08%
Patients and caregivers are advised about oral implications of medications	29	30.53%
Mouthcare is incorporated into care planning for all patients	48	50.53%
Mouthcare is documented to the same level of detail as other care responsibilities	36	38.30%
Prioritization of mouthcare among ADLs (Activities of Daily Living) (Responded High or very high)	48	50.53%
Percieved barriers to performing daily mouthcare (multi-select question)		
Lack of patient cooperation	80	87.91%
Patient unstable/critically ill	67	73.63%
Patient's competing medical needs	59	64.84%
Caregiver/patient refusal	46	50.55%
Lack of time	43	47.25%
Unable to access the mouth	38	41.76%
Lack of an assessment tool/guideline	14	15.38%
Lack of available oral hygiene aides	13	14.29%
None	1	1.10%
Approaches when experiencing challenges performing daily mouthcare (multi-select question)		
Try again at another time	66	71.74%
Reapproach and address challenges in the moment	34	36.96%
Make a note and address with the care team	34	36.96%
Don't provide daily mouth care	11	11.96%
Never experienced challenges	2	2.17%

* Response rate varies per question.

The majority (57%, *n* = 52) of participants did not recall receiving formal oral care education. Among the 43% (*n* = 40) of participants who reported receiving oral care education within their respective training program, almost half (42%, *n* = 17) felt it was inadequate. When asked about oral health continuing education opportunities, most nurses (87%, *n* = 80) expressed interest in the topic. (Table 2).

A statistically significant difference was identified regarding the participant’s knowledge of the recommendation of a pea-sized amount of toothpaste for a child aged 3 years and above between nurses with 5 or fewer years of experience and 6 or more years of experience, *Χ*^2^ (1, *N* = 96) = 4.101, *p* = .043) with a Cramer's V of (.207). There were no differences in knowledge regardless of age, years of experience or exposure to oral health curriculum with nursing programs (Table 3).

**Table 3:** Chi square analysis of survey results.

Children over 3 and able to spit can use a pea sized amount of fluoridated toothpaste						
By experience	Reponded correctly	Responded incorrectly	Total	df	*X^2^*	*p*
0-5 years	35	6	41	1	0.207	0.043
6+ years	37	18	55			
Total	72	24	96			
By Unit						
Intensive Care Unit	13	10	23	4	0.329	0.036
Medical Unit	22	5	27			
Medical, Surgical and Neurosciences Unit	11	0	11			
Nephrology, Hematology and Oncology Unit	13	7	20			
Psychiatric Inpatient Unit	12	2	14			
Total	71	24	95			
Patient's oral health status is routinely assessed on admission						
By Unit	Responded yes	Responded no or I don't know	Total	df	*X^2^*	*p*
Intensive Care Unit	4	19	23	4	0.555	<.001
Medical Unit	3	24	27			
Medical, Surgical and Neurosciences Unit	2	9	11			
Nephrology, Hematology and Oncology Unit	15	5	20			
Psychiatric Inpatient Unit	2	12	14			
Total	26	69	95			
Patients and caregivers are advised about oral implications of medications						
By exposure to formal oral health curriculum	Responded yes	Responded no or I don't know	Total	df	*X^2^*	*p*
Yes	22	18	40	2	0.446	<.001
No	7	45	52			
Total	29	63	92			
By Unit						
Intensive Care Unit	4	19	23	4	0.405	0.004
Medical Unit	5	22	27			
Medical, Surgical and Neurosciences Unit	1	9	10			
Nephrology, Hematology and Oncology Unit	9	11	20			
Psychiatric Inpatient Unit	9	5	14			
Total	28	66	94			
Mouthcare is incorporated into care planning for all patients						
By experience	Responded yes	Responded no or I don't know	Total	df	*X^2^*	*p*
0-5 years	13	28	41	1	0.328	0.001
6+ years	35	19	54			
Total	48	47	95			
By Unit						
Intensive Care Unit	21	2	23	4	0.516	<.001
Medical Unit	10	17	27			
Medical, Surgical and Neurosciences Unit	4	6	10			
Nephrology, Hematology and Oncology Unit	10	10	20			
Psychiatric Inpatient Unit	2	12	14			
Total	47	47	94			
Mouthcare is documented to the same level of detail as other care responsibilities						
By Unit	Responded yes	Responded no or I don't know	Total	df	*X^2^*	*p*
Intensive Care Unit	16	6	22	4	0.390	0.007
Medical Unit	7	20	27			
Medical, Surgical and Neurosciences Unit	3	7	10			
Nephrology, Hematology and Oncology Unit	6	14	20			
Psychiatric Inpatient Unit	4	10	14			
Total	36	57	93			
Prioritization of mouthcare among ADLs (Activities of Daily Living)						
By experience	Responded high or very high	Responded moderate or low	Total	df	*X^2^*	*p*
0-5 years	14	27	41	1	0.285	0.005
6+ years	34	20	54			
Total	48	47	95			
By Unit						
Intensive Care Unit	17	6	23	4	0.328	0.039
Medical Unit	12	15	27			
Medical, Surgical and Neurosciences Unit	3	7	10			
Nephrology, Hematology and Oncology Unit	7	13	20			
Psychiatric Inpatient Unit	9	5	14			
Total	48	46	94			

### 3.3 Attitudes and prioritization

Oral care was ranked as a high or very high priority among the activities of daily living (ADLs) by most participants (51%, *n* = 48), and almost all participants (94%, *n* = 89) agreed or strongly agreed oral care is equally important for all patients. However, only 27% (*n* = 25) felt oral care is performed as often as necessary. (Table 2) Among ADLs, nurses with more than five years of experience prioritized mouthcare higher than those with five years of experience or less [*Χ*^2^ (1, *N* = 95) = 7.742, *p* = .005]) with a Cramer's V of (.285). As well, nurses who worked in the Intensive Care Unit prioritized mouthcare higher than Nurses working in the Medical Unit, Medical, Surgical and Neurosciences Unit, Nephrology, Hematology and Oncology Unit or the Psychiatric Inpatient Unit (Table 3).

Most participants believed oral care is a shared responsibility between the caregiver/patient and the health provider. Eighty two percent of participants (*n* = 76) agree that it is their responsibility to ensure oral care is delivered either by the patient/caregiver or by themselves, while most (76%, *n* = 71) also believe it is the responsibility of the patient or their caregiver to ensure oral care is delivered, either by asking for assistance or completing it themselves (Table 2).

### 3.4 Practices

This study found that only 27% (*n* = 26) of participants reported that oral health status is assessed on admission (Table 2). Furthermore, a comment in an open-ended text box suggested that most patients perform mouthcare themselves or have a caregiver assist and nurses do not observe or assess the adequacy of plaque control. There are statistically significant and strong differences in assessment practices between units. The Nephrology, Hematology and Oncology Unit were most likely to assess the oral cavity, *Χ*^2^ (4, *N* = 95) = 29.241, *p* = <.001) with a Cramer's V of (.555) (Table 3).

Only 31% (*n* = 29) of Nurses responded that patients and caregivers are advised about oral implications of medications. (Table 2) There were statistically significant and strong differences in whether oral implications of medications were advised upon between nurses who experienced oral health curriculum in their formal nursing education and those who did not, *Χ*^2^ (2, *N* = 92) = 18.340, *p* = <.001) with a Cramer's V of (.446). A comparison between units revealed that the Nephrology, Hematology and Oncology and Psychiatric Inpatient Units were the most likely to have this conversation with patients and caregivers, *Χ*^2^ (4, *N* = 94) = 15.381, *p* = .004) with a Cramer's V of (.405) (Table 3).

Just over half of the Nurses (51%, *n* = 48) reported that mouthcare is incorporated into care planning for all patients. (Table 2) Nurses working in the Intensive Care Unit and those with six or more years of experience were most likely to incorporate mouthcare into care planning for all patients, *Χ*^2^ (4, *N* = 94) = 25.053, *p* = <.001) with a Cramer's V of (.516) and *Χ*^2^ (1, *N* = 95) = 10.219, *p* = <.001) with a Cramer's V of (.328) respectfully.

Documentation of mouthcare at a comparable level of detail as other care responsibilities was reported by only 38.30% (*n* = 36) of Nursing Staff. (Table 2) Detailed documentation of mouthcare was reported most often by Nurses working in the Intensive Care Unit, *Χ*^2^ (4, *N* = 93) = 14.156, *p* = .007) with a Cramer's V of (.390) (Table 3).

### 3.5 Barriers

When asked what is preventing daily mouthcare from being delivered to patients, commonly reported barriers included the patient's uncooperative behaviour, the patient being unstable or critically ill, competing medical needs, patient or caregiver refusal, lack of time, unable to access the mouth, lack of an assessment tool/guide and lack of available oral hygiene aides (Table 2). In the open-ended responses, suction toothbrushes were a commonly reported aide that the Nurses desired for managing intubated patients and patients who have trouble swallowing.

## 4 Discussion

This study identified discrepancies between oral health knowledge, attitudes, and practices of pediatric nursing staff. Most nurses viewed oral health as a significant component of overall health and most agreed they are comfortable in assessing the mouth and performing daily mouthcare; yet nurses acknowledge that oral care is not performed as often as necessary for hospitalized children. These findings are similar to those previously reported by Costello and Coyne (19). Further supporting these findings was Adib-Hajbaghery et al. who found that for ventilated patients, oral care was given a moderate ranking of 5.7 on a Likert scale of 1–10 for priority, while more than 21% of nurses did not perform oral care as part of their patient routines (20). These findings demonstrate a discrepancy between the knowledge, attitudes and practices of nurses regarding oral care. This study also found that there are statistically significant and strong differences in the prioritization of oral health based on years of experience. This finding may be attributed to new nurses feeling overwhelmed with required duties, whereas experienced nurses have improved time management allowing them more time to prioritize oral health.

This study found that the majority (82%, *n* = 76) of participants feel it is their responsibility to provide oral care and the responsibility of the patient or caregiver to ensure oral care is performed (76%, *n* = 71). This likely attributed to oral care being underperformed in the hospital setting. Further research is needed to explore how this entrusted responsibility is delineated and communicated and to gain the caregiver's perspective. Furthermore, only 51% (*n* = 48) report incorporating oral care into the care plans for all patients. Oral care plans with input from the health care team and patient's families best address the patient's unique needs (25). Developing an oral care plan for each patient on admission will address the ambiguity of who is responsible for performing oral care (e.g., patient, caregiver, nurse) and identify and plan to overcome barriers to care.

This study found that the Nephrology, Hematology and Oncology Unit are more likely to assess the oral cavity than other inpatient units. This finding may be attributed to their long-term relationship with the Dental Department and existing guidelines requiring every patient diagnosed with a cancer to receive a dental assessment at time of diagnosis. This study also found that the Intensive Care Unit was more likely to discuss oral health implications, and plan and document oral care. These findings may be due to the systemic complications that can arise from intubation such as ventilator- associated pneumonia and trauma to the oral cavity. In addition, Nurses working in the Psychiatric Inpatient Unit were also likely to discuss oral health implications with patients and caregivers. This is likely a result of the oral health implications commonly found in medications used to treat psychiatric conditions, such as xerostomia (perceived dry mouth) and hyposalivation (low salivary flow).

The availability of oral care supplies was a practical barrier for nurses. In the open-ended text boxes, study participants described a lack of quality and appropriately sized toothbrushes, as only small child sized toothbrushes were routinely stocked. They also reported the lack of available child-friendly toothpaste with mild flavours as a barrier. The frequent use of oral care sponge swabs was reported in this study. This finding was not unexpected as oral care sponge swabs are more available within the inpatient units and other studies have reported similar preferences to stock oral care sponges rather than quality toothbrushes (19, 26). However, oral care sponge swabs are not as effective at removing plaque when compared to toothbrushing and can be damaging to oral mucosa when used inappropriately (27). The development of a pediatric hospital guideline outlining best practice assessment tools, oral care plans and proper use of oral care tools is needed.

The results of this study highlight the need for further integration of oral care into nursing practice within the pediatric hospital setting. To support the integration of oral care, the use of an oral health program guideline is supported by the World Health Organization and should include oral assessment, daily mouth care, referrals (as needed) and continued education, including oral hygiene instruction and dietary counselling (6). Oral health program guidelines must be based on current evidence, include input from stakeholders and address the barriers to oral care that exist within the institution. To further integrate oral care into the culture of inpatient units, an interdisciplinary approach including oral health care professionals should be considered (25). More research is needed to explore how this entrusted responsibility is delineated and communicated between caregivers and care providers and the caregiver’s perspective regarding oral care for their hospitalized family member. Also of interest is exploration of the patient, care provider and caregiver factors prompting or contributing to an oral assessment, oral care plan and documentation. Lastly, based on these findings, future research includes a randomized prospective intervention study to determine the effectiveness of an oral health care guideline in a hospital setting and the impact on the oral health of hospitalized children.

Lack of formal oral health education for nurses may contribute to underperformed oral care. The study showed significant differences between exposure to formal oral health curriculum and whether oral health implications of medication were discussed demonstrating the need for oral health in nursing curriculum. There was a lack of knowledge regarding the basic etiology of dental caries, as half of the respondents did not know dental caries was an infectious disease and two-thirds were unaware that cariogenic bacteria are transmissible. It is encouraging, however, that eighty-three percent of participants were interested in further oral health education, including specialized oral care for critically ill children and managing responsive behaviours during oral care, as suggested in the open-ended responses. Moreover, the need for strategies to improve cooperation was also identified in this study when nursing staff reported lack of cooperation as a common barrier. Education accompanying a pediatric hospital guideline is recommended to improve oral health assessment and oral care practices for hospitalized children.

This study is not without limitations and despite capturing the responses of almost half the target population, no CTA's responded. Therefore, the responses may not be representative of the entire population who provide oral care to inpatients, resulting in inaccurate profiling of oral care practices in paediatric hospitals. Survey responses are inherently subject to social desirability bias and nursing staff may have selected responses that reflect their practice ideals rather that the current state. Survey responses were limited to nurses in a single pediatric hospital and may not reflect knowledge, attitudes, and practices at other institutions. Furthermore, the nature of the inpatient units varies with long-term and short-term care, and length of stay within an inpatient unit was not accounted for. Further research is also needed to explore the unique oral care needs and the role of nurses working in neonatal and outpatient care.

In conclusion, maintaining optimal oral health for hospitalized children is essential to improve their general health and quality of life. Nurses value the importance of oral health for hospitalized children and play a vital role in providing daily oral care. Barriers to providing oral health care in a hospital setting have been identified as patient being uncooperative, critically unstable, competing medical needs, patient/caregiver refusal, time, difficult access to mouth, and lack of assessment tools and oral hygiene aides. Further research is needed to fully understand pediatric hospital nurses’ current practices and the many potential patient-specific factors that exist. Each reported barrier to providing oral care is important to investigate and address. Further continuing education related to oral health was clearly identified as an important first step. The development of institution specific oral care guidelines, and interprofessional support from oral health care professionals are further recommendations to improve the oral health of hospitalized children.

## Data Availability

The original contributions presented in the study are included in the article/Supplementary Material, further inquiries can be directed to the corresponding author.
